# Accuracy of five different 3D printing workflows for dental models comparing industrial and dental desktop printers

**DOI:** 10.1007/s00784-022-04809-y

**Published:** 2022-12-03

**Authors:** Belén Morón-Conejo, Jesús López-Vilagran, David Cáceres, Santiago Berrendero, Guillermo Pradíes

**Affiliations:** 1 Department of Conservative and Prosthetic Dentistry, Faculty of Dentistry, University Complutense of Madrid, Plaza Ramón Y Cajal S/N. 28040, Madrid, Spain; 2 Clínica Dental Vilagran, Badalona, Barcelona, Spain

**Keywords:** 3D impression, Dental models, Additive manufacturing, Industrial printer, Dental desktop printer

## Abstract

**Objectives:**

The aim of this study was to evaluate the accuracy, in terms of trueness and precision, of printed models using five different industrial and dental desktop 3D printers.

**Materials and methods:**

Full-arch digital models with scanbodies of 15 patients were printed with five different 3D printers. The industrial printers were 3D system Project MJP2500 (3DS) and Objet30 OrthoDesk (Obj). The dental desktop printers were NextDent 5100 (ND), Formlabs Form 2 (FL) and Rapidshape D30 (RS). A total of 225 printed models were analysed. The printed models were digitized and compared with the reference cast model using the Control X software (Geomagic). The descriptive statistics and one-way ANOVA with the post hoc Tukey test were performed (*α* = 0.05).

**Results:**

The one-way ANOVA for the trueness and precision of the printed model presented the best results for the 3DS, followed by ND, Obj, FL and RS (*P* < 0.01). In the scanbody zone, the best results were for the 3DS group, followed by Obj, ND, FL and RS (*P* < 0.01). Comparing the technologies, the Multijet technology used in industrial printers presented better results than the DLP and SLA technologies used in dental desktop printers (*P* > 0.01).

**Conclusions:**

There were statistically significant differences in terms of the accuracy of the printed models, with better results for the industrial than the dental desktop 3D printers.

**Clinical relevance:**

The industrial 3D printers used in dental laboratories presented better accuracy than the in-office dental desktop 3D printers, and this should be considered when the best accuracy is needed to perform final prosthetic restorations.

## Introduction

In recent years, dentistry has undergone a digital revolution, and computer-aided design and computer-aided manufacturing (CAD/CAM) technology is now a daily practice in any field of dentistry. Using an extraoral or intraoral scanner, we obtain an STL file with the three-dimensional (3D) data of our patients’ mouths [1–3]. After that, with various CAD software available and supported by the dental technician, the dentist obtains a range of devices, such as surgical guides, orthodontics splints, prosthetic structures (crowns or bridges) and models, among others [4]. To move from the virtual to the real environment, designs must be manufactured using CAM systems [5]. In this step, a fast-growing alternative to milling methods is 3D printing, also called additive manufacturing or rapid prototyping. This technology allows the fabrication of objects layer by layer in cross-sections from digital designs or impressions [6, 7]. 3D printing manufacturing, compared to subtractive methods, allows the construction of objects with complex geometries, and no material is wasted, leading to a more sustainable process [8]. In addition, there are processes that allow for working with different raw materials, which may be grouped into binder/powder material combinations, including polymers (resins and thermoplastics), metals and ceramics [7].

One of the most common applications for 3D printing using polymeric materials is producing dental models. There are different types of 3D printers depending on the technology used [9, 10]. The American Society for Testing and Materials (ASTM) divides the available 3D printing technologies into seven categories, ISO/ASTM 52,900: 2015 [11, 12], three of which are the most popular for generating dental models: stereolithography (SLA), material jetting (Multijet) and material extrusion or fused deposition modelling (FDM). The SLA category includes digital light processing (DLP). SLA and DLP have similarities since the objects are built layer by layer by immersing a build platform in a resin tank containing light-cured liquid resin [13]. The key difference between these two technologies is the type of light source: SLA uses an ultraviolet (UV) laser light to draw a pattern of a cross-section of the 3D object [14, 15], and DLP uses a digital light projector screen to project the entire cross-section of the 3D object at once [16]. Multijet or material jet technology uses materials extruded from nozzles or photopolymers jetted over the workspace, and then the object is polymerized with a UV light source [9, 13]. FDM technology builds parts layer-by-layer from the bottom up by heating and extruding a thermoplastic filament from a printing nozzle. The nozzle repeats the extruding and melting layer by layer until the object is complete [17, 18]. The industrial 3D printers that are used in the dental field use Multijet, whereas the dental desktop 3D printers available use SLA or DLP technology. The scientific literature in the dental field mainly analyses printers specifically designed for dental use. These dental 3D printers are smaller in terms of volume and cheaper due to their simplified building technology and lower resolution and velocity than industrial 3D printers.

In addition to the printing technology, many other factors can influence the results of the printed model, such as the type of material, layer thickness, depth of cure, build orientation, platform position, amount of support structures and postprocessing procedure [19]. Therefore, the accuracy of a printed model is influenced by many factors. According to the ISO, accuracy consists of two parameters: trueness and precision. (ISO 5725–1: 1994, ISO 12836: 2015) [20, 21]. The trueness of a 3D printer is described as the deviation of the printed object from its actual dimensions, and the precision of a 3D printer is the deviation between repeated prints [22–24]. High trueness describes the proximity of the original dimensions of the measured object, and high precision defines a 3D printer´s ability to manufacture the same product with the same dimensions in repetitive prints [25].

To date, the number of studies related to the accuracy of 3D-printed working models is limited, and no differences between industrial or dental desktop printers are normally considered. Therefore, the aim of this study was to evaluate the accuracy, in terms of precision and trueness, of the physical models obtained after digitization with an intraoral scanner of the maxillary arch of patients using different 3D printing media.

The proposed null hypothesis was that there would be no statistically significant differences in terms of accuracy, expressed as precision and trueness, of the physical models obtained after digitization with an intraoral scanner of the full-arch of patients using different 3D printing technologies, both industrial and in office dental desktop printers.

## Materials and methods

The study protocol was approved by the Ethics Committee of the Hospital Clinic San Carlos in Madrid (C.P. AVINENT – C.I. 21/484-E) and followed the ethical principles established in the Declaration of Helsinki. A total of 15 patients with 15 Biomimetic Ocean IC single implants (Avinent) gave their informed consent to use their models for the in vitro study.

### Reference models and digital impressions

A conventional impression with heavy and light silicone using an open tray transfer (Avinent IC, ref. 0475 + 0480) was obtained from each patient. A cast model (FujiRock EP, GC, Japan) with the implant analogue (Avinent IC, ref. 0585) was obtained the day after the impression in the dental lab. Then, each cast model was scanned using an extraoral scanner (3shape lab scan Model D2000) with an accuracy (ISO128/36) [21] of 5 microns. The digital models obtained with the extraoral scanner from the cast models were used as the reference model (RM) of each patient.

Subsequently, a digital impression with the Trios 3 intraoral scanner (3Shape) and the corresponding scanbody Avinent IC (ref. 2801, Avinent) of the implant was obtained from each patient. Digital impressions were obtained following the manufacturer’s instructions and were performed by the same operator (J.V.), starting with posterior occlusal from distal to mesial, making vestibule-palatine movements in the anterior area, following the palatal or lingual zone, and finally going through the vestibular part. The operator used an OptraGate (Ivoclar Vivadent) for retraction of the lips and avoided any direct impact of the equipment light during the digital impression with the intraoral scanner.

### 3D impression of the study samples

In this in vitro study, the industrial 3D printers with Multijet technology were the 3DS Projet MJP2500 (3DS group) and Objet30 OrthoDesk (Obj group). The dental desktop 3D printers were NextDent 5100 (ND group) and Rapidshape D30 (RS group) with DLP technology and Formlabs Form 2 (FL group) with SLA technology (Table 1).

**Table 1:** Study groups printer characteristics

Group	Printer	Technology	Resin	Layer thickness
3DS	3D system Projet MJP 2500	Multijet	VisiJet M2 SUP	32 µ
Obj	Objet30 OrthoDesk	Multijet	Support SUP705	30 µ
ND	NextDent 5100	DLP	Model 2.0 Peach	50 µ
FL	Formlabs Form 2	SLA	Dental Model Peach	50 µ
RS	Rapidshape D30	DLP	Model 2.0 Peach	25 µ

The digital impressions were sent to the Avinent CAD/CAM Centre (Barcelona, Spain), where the split cast of the digital impression prior to 3D printing was designed with Model Builder software (vs. 2019). The 3D nesting programs recommended by the manufacturer of each study group were used following the protocol for the model printing (3D sprint basic in the 3DS group, Objet studio in the Obj group, 3D sprint basic in the ND group, Preform in the FL group and Netfabb Professional in the RS group). The models were printed with the dental model resin and layer thickness recommended for each 3D printer workflow (Table 1). All of the models were hollow and placed horizontally with a 0° inclination to the platform. The total sample size was 225 printed models, 45 models per study group obtained from printing the patient’s digital models 3 times with each printer.

The postprocessing protocol was the same in the ND, RS and FL groups, where the models were cleaned with C3H8O and air-dried for 30 min. In the 3DS group, the models were cleaned with pressurized water, and in the Obj group, they were cleaned with steam, oil and pressurized water. The curing times were between 20 and 60 min at 60 °C following the manufacturer’s indications.

### Analysis and comparison of the study samples

**Fig. 1:**
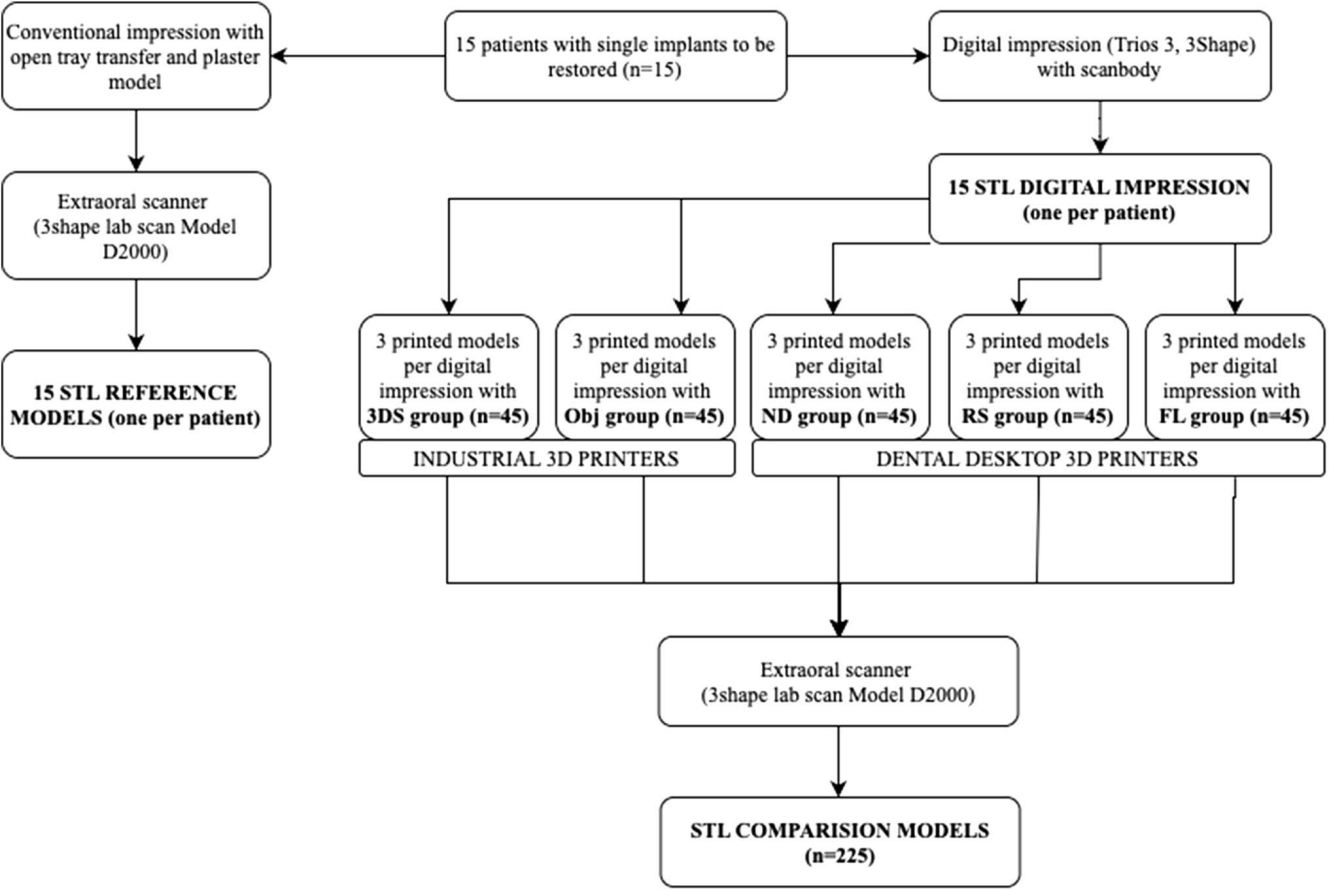
Flow chart of the research design. Legend: Study groups: 3DS, 3D system Projet MJP 2500; Obj, Objet30 OrthoDesk; RS, Rapidshape D30; FL, Formlabs 2; ND, NextDent 5100

The 225 printed models were digitized with the same extraoral scanner as the RM of the patients (3Shape LabScan Model D2000) (Fig. 1). Due to the known shrinkage and deformation of the resins used with 3D printing, the time of analysis and digitalization of the printed models was 10 days after the postprocessing protocol was finished. Subsequently, the digital files were analysed using Control X software (vs. 2018.1.0., Geomagic, 3D systems). The alignment and superposition of the models were performed in two phases: an *initial alignment* with volume recognition and in the second alignment with a *best fit algorithm* to obtain the maximum adjustment of the surfaces. One of the limitations of the best fit algorithms is that it tends to underestimate the real discrepancies found when searching for the maximum fit between the surfaces.

The first comparison of the full-arch models and their global surface was made within a discrepancy of less than 1 mm and a measurement tolerance selected in ± 100 microns (µm). A second comparison selecting only the scanbody zone was performed to study the scanbody discrepancies with the 3D-printed surface using the same analysis parameters (Table 2). The scanbody area selected for comparison was the entire cylinder volume, and in the case of the RM, the scanbody was screwed on the implant analogue prior of its digitization. Each of the 3D-printed models was compared with the RM of each patient to obtain the intergroup results and the trueness. The intragroup values analysed the precision of each study group, comparing the printed models of each study group. A colour scheme range was obtained where positive discrepancy values (yellow to red) depicted areas where the printed model was larger than the RM, and negative values (turquoise to blue) presented areas where the printed model was undersized.

**Table 2:** Statistical variables

Variable	Description
Trueness (RMS mean deviations)	Mean average distance of all points from each STL to RM in absolute values
Precision (standard deviation of RMS)	Standard deviation of the distances between points of each STL and RM in the RMS values
External mean discrepancy (over total %)	Percentage of the STL model that is oversized from the RM
Internal mean discrepancy (lower total %)	Percentage of the STL model that is undersized from the RM

Legend: *RMS* root mean square, *STL* standard triangle language file, *RM* reference model

### Statistical analysis

Descriptive statistics (mean values, standard deviations, medians and 95% confidence intervals) of variables were calculated for each group using a statistical software program (SPSS version 22.00). The sample size was calculated with an effect size of 0.55 with a standard deviation (SD) of 0.035, an alpha error of 0.05 and a statistical power of 90%. The resulting sample size was at least 70 samples with 14 samples per group. Finally, our final sample size was 225 samples, 45 printed models per group with 3 printings of each 15-patient digital model. To assess the reliability data, the precision and trueness of the models were obtained by comparing the groups using the ANOVA test with the post hoc Tukey test and nonparametric analysis (median test) with a significance level of 95%. The median test was performed to analyse the external and internal mean discrepancies.

## Results

### Trueness and precision of the full-arch printed model of each study group (Fig. 2)

**Fig. 2:**
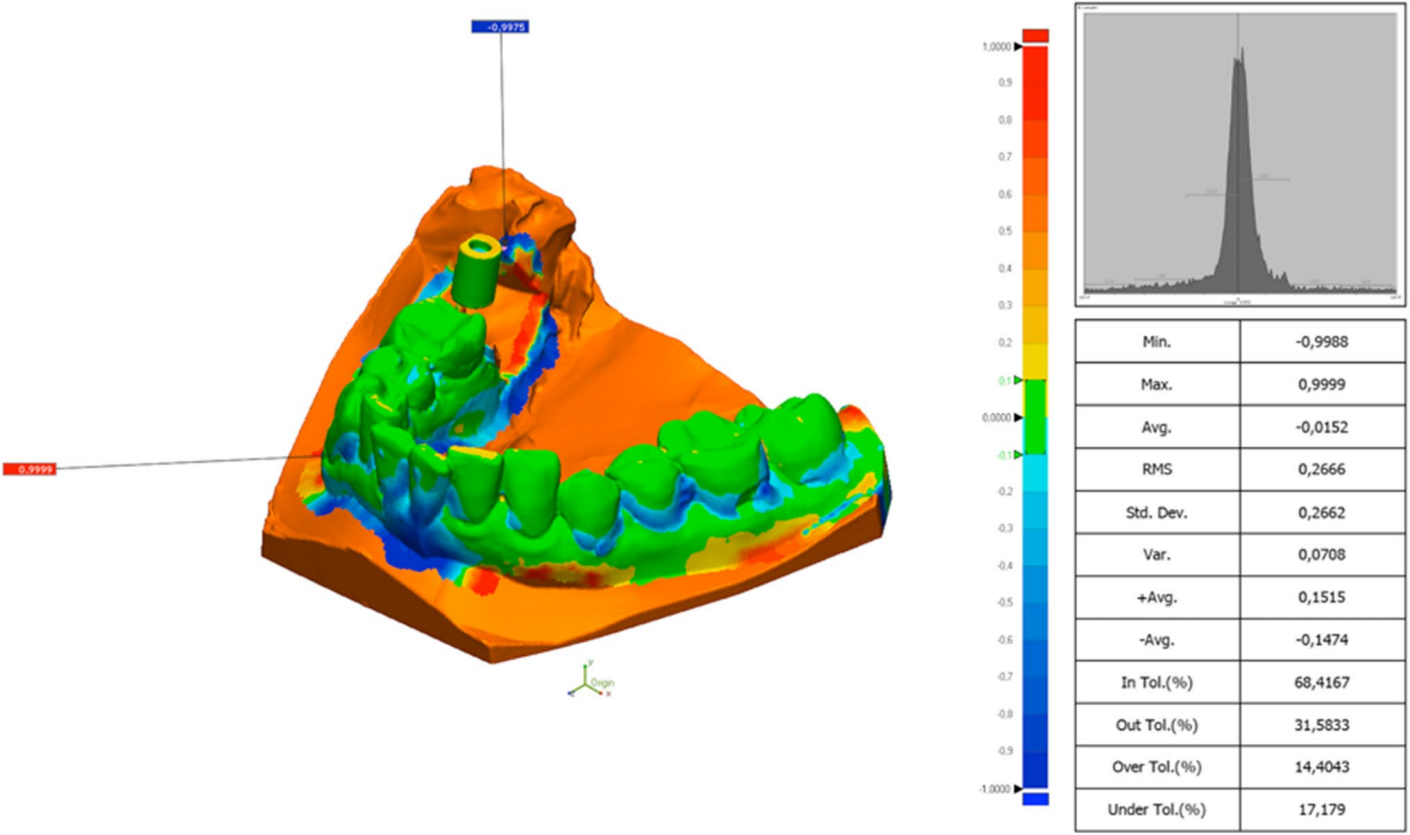
Colour scheme range of the comparison between the STL of a printed model and its reference full-arch model with Geomagic Control X (3D System). Legend: the units are in millimetres

**Fig. 3:**
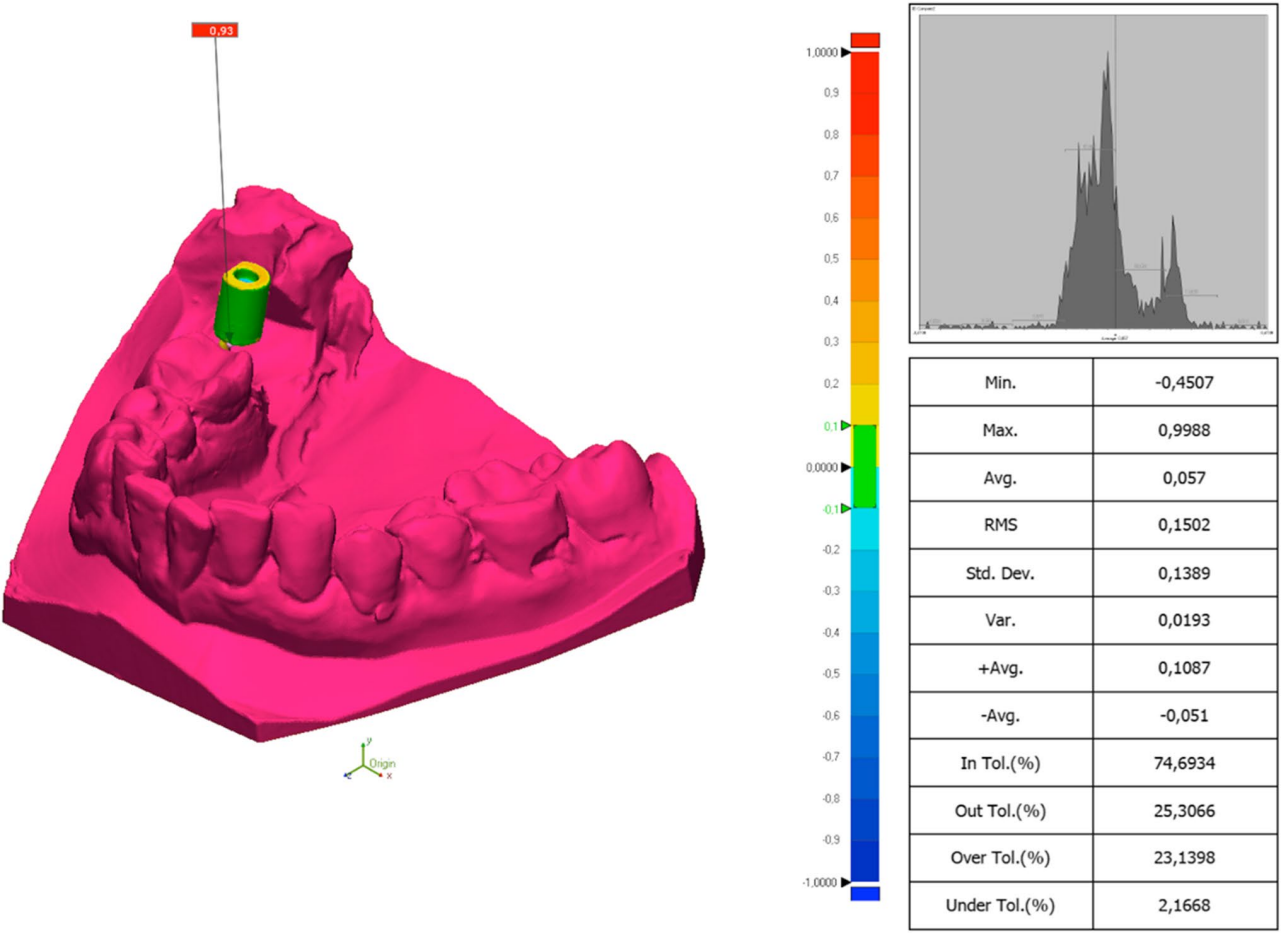
Colour scheme range of the comparison between the STL of a printed model and its reference model in the scanbody zone with Geomagic Control X (3D System). Legend: the units are in millimetres

**Fig. 4:**
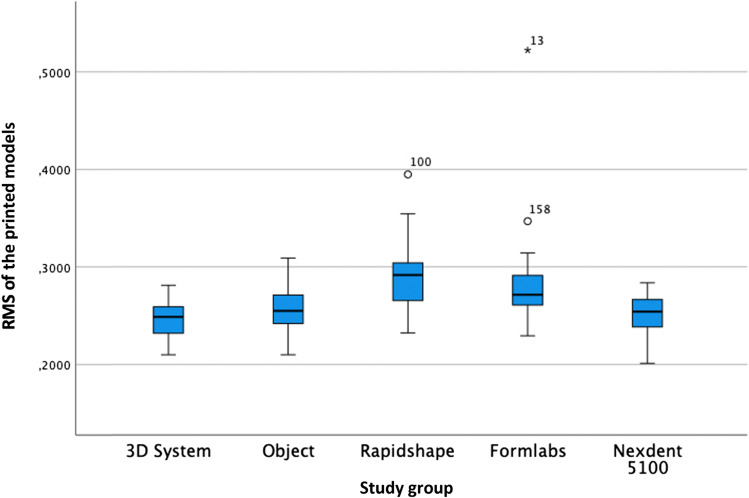
Boxplot of the RMS of the printed models

According to the trueness of the printed model, the RMS one-way ANOVA test was statistically significant (*F* = 27.094; *P* < 0.01) (Table 3 and Figs. 3 and 4). Although the RMS values have been used for the statistical evaluation, the linear mean values have also been reflected for analysis and comparison with other research groups. The post hoc Tukey test showed that the best trueness was obtained by the 3DS group, followed by ND, Obj, FL and RS.

**Table 3:** Trueness of the model analysing the RMS (root mean square) and linear measurements (LM) in µm

Study group	Mean	Median	SD	*P* value	Post hoc Tukey test RMS
3DS	RMS 245.05 LM 140.13	RMS 248.80 LM 138.70	RMS 18.38 LM 17.28	< 0.01*	Obj *P* = 0.599 RS *P* = 0.000* FL *P* = 0.000* ND *P* = 0.945
Obj	RMS 255.25 LM 157.12	RMS 255.08 LM 155.90	RMS 22.53 LM 19.13	< 0.01*	3DS *P* = 0.599 RS *P* = 0.000* FL *P* = 0.001* ND *P* = 0.982
RS	RMS 288.58 LM 200.83	RMS 291.70 LM 201.10	RMS 33.93 LM 31.40	< 0.01*	3DS *P* = 0.000* Obj *P* = 0.000* FL *P* = 0.001* ND *P* = 0.000*
FL	RMS 281.47 LM 193.71	RMS 271.50 LM 183.30	RMS 45.62 LM 47.95	< 0.01*	3DS *P* = 0.000* Obj *P* = 0.001* RS *P* = 0.875 ND *P* = 0.000*
ND	RMS 250.83 LM 152.63	RMS 254.20 LM 158.6	RMS 20.75 LM 19.25	< 0.01*	3DS *P* = 0.945 Obj *P* = 0.982 RS *P* = 0.000* FL *P* = 0.000*

Legend: * for statistically significant differences (*P* < 0.001) and *SD* standard deviation. Study groups: *3DS* 3D system Projet MJP 2500, *Obj* Objet30 OrthoDesk, *RS* Rapidshape D30, *FL* Formlabs 2, *ND* NextDent 5100

**Fig. 5:**
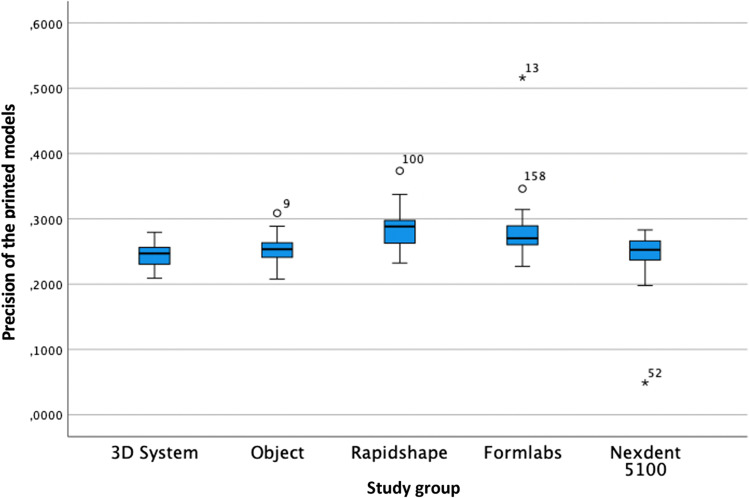
Boxplot of the precision of the printed models

The analysis of the precision of the printed models with one-way ANOVA was statistically significant (*F* = 23.125; *P* = 0.001) (Table 4 and Fig. 5) when analysing the standard deviation of the root mean square (RMS). According to the post hoc Tukey test, the best precision of the printed models was obtained by the 3DS group, followed by ND, Obj, FL and RS.

**Table 4:** Precision of the printed models in µm

Study group	Mean	Median	SD	*P* value	Post hoc Tukey test
3DS	243.88	247.10	18.27	< 0.01*	Obj *P* = 0.827
					RS *P* = 0.000*
					FL *P* = 0.000*
					ND *P* = 1.000
Obj	252.15	253.60	21.44	< 0.01*	3DS *P* = 0.827
					RS *P* = 0.000*
					FL *P* = 0.001*
					ND *P* = 0.888
RS	284.34	288.40	29.50	< 0.01*	3DS *P* = 0.000*
					Obj *P* = 0.000*
					FL *P* = 0.992
					ND *P* = 0.000*
FL	280.34	270.30	44.94	< 0.01*	3DS *P* = 0.000*
					Obj *P* = 0.001*
					RS *P* = 0.992
					ND *P* = 0.000*
ND	244.81	252.70	38.12	< 0.01*	3DS *P* = 1.000
					Obj *P* = 0.888
					RS *P* = 0.000*
					FL *P* = 0.000*

Legend: * for statistically significant differences (*P* < 0.001) and *SD* standard deviation. Study groups: *3DS* 3D system Projet MJP 2500, *Obj* Objet30 OrthoDesk, *RS* Rapidshape D30, *FL* Formlabs 2, *ND* NextDent 5100

### Trueness and precision of the scanbody zone of each study group (Fig. 3)

**Fig. 6:**
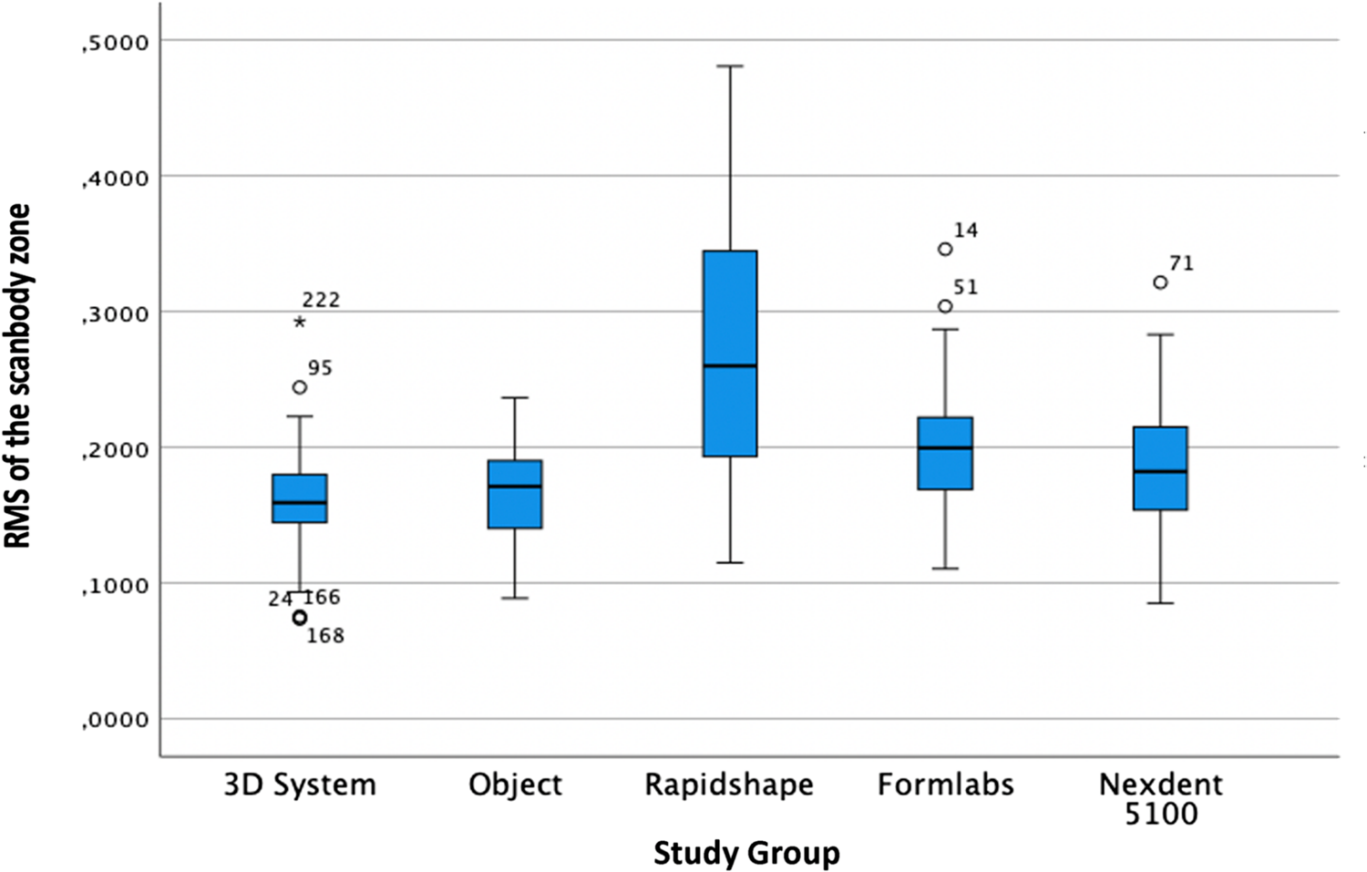
Boxplot of the RMS of the scanbody zone

According to the trueness of the scanbody, the RMS one-way ANOVA test was significantly different (*F* = 48.258; *P* < 0.01) (Table 5 and Fig. 6). Although the RMS values have been used for the statistical evaluation, the linear mean values have also been reflected for analysis and comparison with other research groups. The post hoc Tukey test showed that the best trueness in the scanbody zone was obtained by the 3DS group, followed by Obj, ND, FL and RS.

**Table 5:** Trueness of the scanbody zone analysing the RMS (root mean square) and linear measurements (LM) in µm

Study group	Mean	Median	SD	*P* value	Post hoc Tukey test
3DS	RMS 161.80 LM 88.00	RMS 159.30 LM 83.05	RMS 44.81 LM 32.31	< 0.01*	Obj *P* = 1.000 RS *P* = 0.000* FL *P* = 0.014 ND *P* = 0.629
Obj	RMS 164.58 LM 99.60	RMS 171.20 LM 97.10	RMS 38.21 LM 32.82	< 0.01*	3DS *P* = 1.000 RS *P* = 0.000* FL *P* = 0.020 ND *P* = 0.712
RS	RMS 272.51 LM 203.28	RMS 260.00 LM 207.00	RMS 91.45 LM 82.95	< 0.01*	3DS *P* = 0.000* Obj *P* = 0.000* FL *P* = 0.000* ND *P* = 0.000*
FL	RMS 200.43 LM 136.25	RMS 199.60 LM 133.15	RMS 49.71 LM 43.84	< 0.01*	3DS *P* = 0.014 Obj *P* = 0.020 RS *P* = 0.000* ND *P* = 0.498
ND	RMS 184.35 LM 123.13	RMS 182.30 LM 113.15	RMS 53.81 LM 49.47	< 0.01*	3DS *P* = 0.629 Obj *P* = 0.712 RS *P* = 0.000* FL *P* = 0.000*

Legend: * for statistically significant differences (*P* < 0.001) and *SD* standard deviation. Study groups: *3DS* 3D system Projet MJP 2500, *Obj* Objet30 OrthoDesk, *RS* Rapidshape D30, *FL* Formlabs 2, *ND* NextDent 5100

**Fig. 7:**
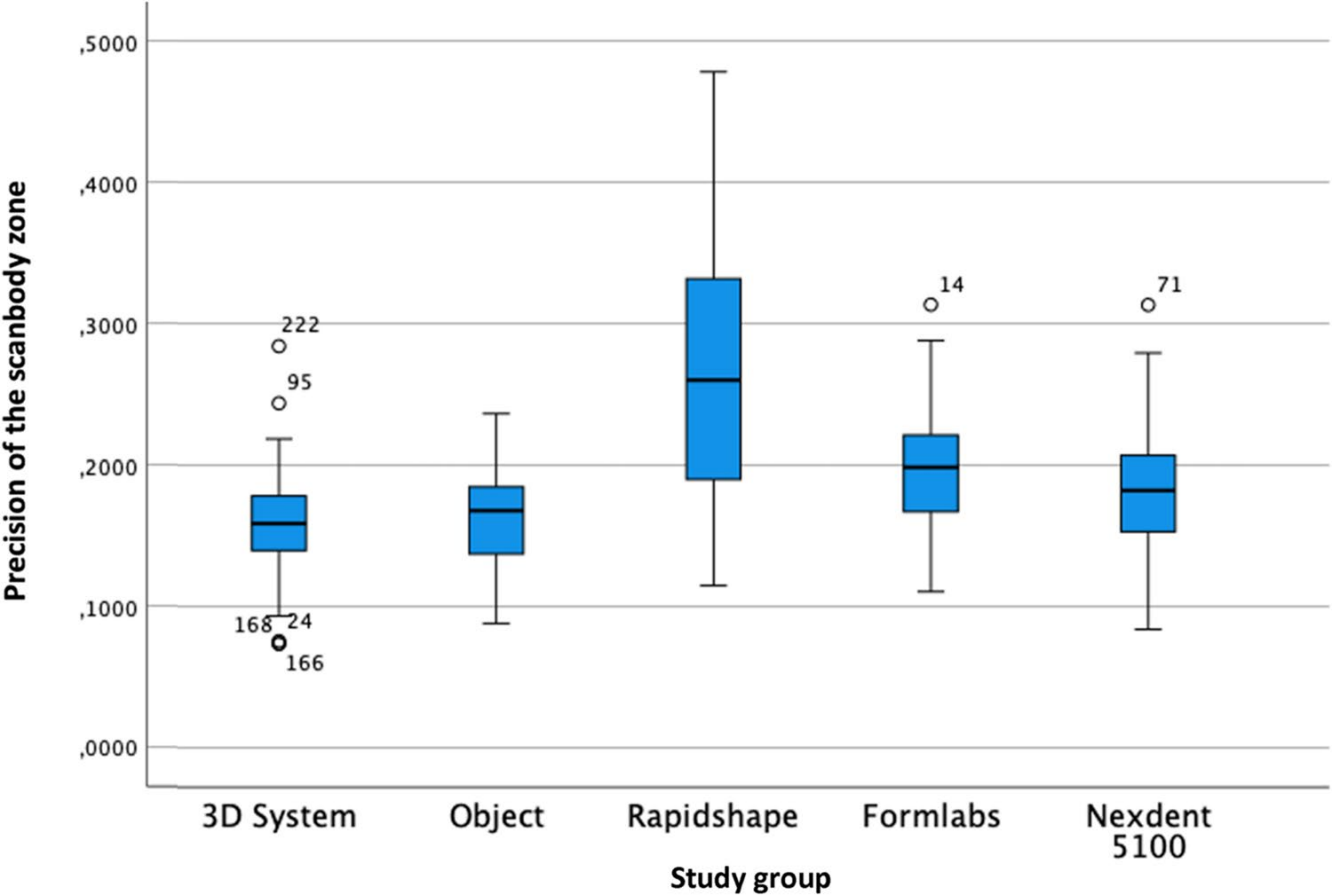
Boxplot of the precision of the scanbody zone

The analysis of the precision of the scanbody zone with the one-way ANOVA test was statistically significant (*F* = 47.305; *P* < 0.01) when analysing the standard deviation of the RMS (Table 6 and Fig. 7). The post hoc Tukey test showed that the best precision in the scanbody zone was obtained by the 3DS group, followed by Obj, ND, FL and RS.

**Table 6:** Precision of the scanbody zone in µm

Study group	Mean	Median	Range	SD	*P* value	Post hoc Tukey test
3DS	159.25	158.70	210.4	43.30	< 0.01*	Obj *P* = 1.000 RS *P* = 0.000* FL *P* = 0.010 ND *P* = 0.565
Obj	162.55	167.80	148.0	37.67	< 0.01*	3DS *P* = 1.000 RS *P* = 0.000* FL *P* = 0.019 ND *P* = 0.686
RS	268.61	260.00	363.3	90.64	< 0.01*	3DS *P* = 0.000* Obj *P* = 0.000* FL *P* = 0.000* ND *P* = 0.000*
FL	197.88	198.60	202.8	46.75	< 0.01*	3DS *P* = 0.010 Obj *P* = 0.019 RS *P* = 0.000* ND *P* = 0.508
ND	182.45	182.30	229.3	52.33	< 0.01*	3DS *P* = 0.565 Obj *P* = 0.686 RS *P* = 0.000* FL *P* = 0.508

Legend: * for statistically significant differences (*P* < 0.001) and *SD* standard deviation. Study groups: *3DS* 3D system Projet MJP 2500, *Obj* Objet30 OrthoDesk, *RS* Rapidshape D30, *FL* Formlabs 2, *ND* NextDent 5100

### Trueness and precision depending on the 3D printer type: industrial vs. dental desktop

The industrial printers are represented by the 3DS and Obj group with the Multijet technology and the dental desktops by the FL groups with SLA technology and the ND and RS groups with DLP technology. After the analysis of the trueness and precision of each study group, they were analysed by comparing their industrial or dental desktop design with ANOVA. The one-way ANOVA test was statistically significant (*P* ≤ 0.01) for all of the groups in terms of trueness (*F* = 27.155) and precision (*F* = 12.153) of the full arch model and in the scanbody zone (trueness *F* = 33.626 and precision *F* = 24.656). The Bonferroni post hoc test defined between which groups the 3DS and Obj groups presented statistically significant differences from the RS (*P* = 0.00) and FL (*P* = 0.01) groups. The RS group with all of the groups and the FL group presented statistically significant differences from all of the groups except for the ND group (*P* = 0.508). According to these results, the 3DS group presented better results, followed by Obj, ND, FL and RS. Multijet printing technology, which is normally used with industrial 3D printers, presented better results than the DLP and SLA technologies used in dental desktop 3D printers.

### Analysis of the volume above or below the printed model

The percentage of the STL model that was above (OVER TOT. %) and below (LOWER TOT. %) the reference once the alignment was carried out was used to analyse the percentage of the printed model that was bigger or smaller than the RM. If the printer tends to print smaller or bigger models than the real one, this could have clinical implications during the prosthesis design and manufacture. For example, if the printed models are larger than the reference model, then the definitive restoration would not fit in the patient’s mouth. Neither the total model nor the scanbody analysis met the normality criteria, so nonparametric tests were used (Kruskal–Wallis test and the median test). The Kruskal–Wallis test showed statistically significant differences among the 5 groups (Table 7). According to the OVER TOT. %, the model analysis 3DS and ND obtained the best results with < 34 and < 29 results under the median. Analysing the scanbody zone, the best results were obtained by 3DS and Obj with < 34 and < 23 results under the median. The best results in the LOWER TOT. % with the model analysis were obtained by Obj and 3DS with < 35 and < 25 results under the median. In the scanbody zone, the best results were obtained with 3DS and Obj with < 40 and < 39 results under the median.

**Table 7:** Frequency analysis by the median test for the over total % and lower total % analysis

	3DS	Obj	RS	FL	ND
% Over tot. model	< 39*	< 7	< 3⊥	< 6	< 29
% Lower tot. model	< 25	< 35*	< 11	< 1⊥	< 11
% Over tot. scanbody	< 34*	< 23	< 13⊥	< 20	< 22
% Lower tot. scanbody	< 40*	< 39	< 6⊥	< 18	< 36

Legend: * for the best result and **⊥** for the worst result. Study groups: *3DS* 3D system Projet MJP 2500, *Obj* Objet30 OrthoDesk, *RS* Rapidshape D30, *FL* Formlabs 2, *ND* NextDent 5100

## Discussion

According to the results of the present study, significant differences in terms of trueness and precision were found between industrial and dental desktop printers. The industrial printers used the Multijet technologies (3DS and Obj groups) and presented lower mean values for trueness and precision (*P* < 0.01) both in the complete-arch model and scanbody surface analysis. Therefore, the null hypothesis that there would be no statistically significant differences in terms of accuracy, expressed as precision and trueness, of the printed models obtained after digitization with an intraoral scanner of the maxillary arch of patients using different 3D printing media, including industrial or dental desktop printers, was rejected.

The results were interpreted taking into consideration the RMS mean deviation values for the accuracy evaluation and its standard deviation for the precision evaluation according to the ISO 5725 standards [20]. This method is frequently used in the scientific literature on this topic [10, 24, 26]. This is the preferred method chosen for the accuracy evaluation as opposed to the average deviation values method, where positive and negative values in the arithmetic mean can neutralize each other and preclude any actual difference. The RMS formula squares them and therefore prevents the neutralization of the opposite signs.

There is great heterogeneity in the scientific literature about 3D printing, and we found it difficult to compare our results to previous studies. The reference scanners used, materials, master models, printing technologies and parameters varied. Analysing the trueness and precision of different 3D printers in full-arch impressions, Kim et al. presented data in concordance with our results, obtaining the best results in terms of precision and trueness of industrial Multijet technology (69 ± 18 μm and 86 ± 17 μm; *P* < 0.05) compared to dental desktop printers with DLP (74 ± 34 μm and 469 ± 49 μm) and SLA technology (176 ± 73 μm and 141 ± 35 μm) [10]. Emir and Ayyildiz in 2021 also obtained statistically significant differences in the precision of the Multijet group (30.4 μm) when compared with the SLA (37.6 μm) and DLP (43.6 μm) groups [24]. In contrast with our results, they reported the highest trueness in the DLP group (46.2 μm) rather than in the Multijet group (58.6 μm), *P* = 0.005. They used a master model digitally designed with cylinders instead of teeth. These simplified geometry characteristics are less challenging to reproduce than a natural dental arch [24]. The analysed results of the scanbody zone of our study showed lower RMS values and better accuracy and precision for all of the study groups and technologies, which could also be explained by its simplified geometry. Even so, it presented the best result with the Multijet technology groups.

In the dental desktop printers, the ones with SLA technology seemed to have higher trueness than the DLP technology for full arch measurements, as it involves a smaller layer thickness and laser point of curing; however, it had lower precision. The DLP technology uses a projector to cure the material layer by layer, reducing the error with repeated impressions. The best results obtained for the Multijet technology could be explained by the new resin cartridge used in each impression and the industrial volume characteristics of the printer [27]. In the SLA and DLP dental desktop printers, the nonpolymerized resin is stored in the printer’s tank, and it was repeatedly used. Industrial printers and Multijet technology can print smaller layer thicknesses than SLA printers, resulting in smoother surfaces and greater detail [28]. Another parameter to consider is the build angle in each 3D printing workflow for different clinical applications, which could influence the dimensional accuracy of 3D-printed restorations [26, 29–31]. The horizontal nesting of the full-arch model is recommended in the 3D printing workflow of the printers used in our study. Nevertheless, some studies have suggested an oblique angle of 30–45° to print the models, as the build angle and layer height presented statistically significant interactive effects on the accuracy of the printed models [26, 29–31]. Another parameter that should be considered in the protocols is the time of analysis, as some studies achieved a lower trueness in models when they were analysed 3 or 4 weeks after printing, suggesting a dimensional contraction of the resin over time [32, 33]. There is a lack of information about this topic in the scientific literature, and more studies are needed about the influence of different parameters in the different 3D printing technologies.

Some authors concluded that 3D-printed models showed the highest RMS mean values in the accuracy (trueness and precision) of the complete arch and the trueness of preparation, although they cannot yet completely replace conventional stone models. A systematic review performed by Etemad-Shahidi in 2020 analysed six DLP printers, five SLA printers and one Multijet printer [28]. All SLA and DLP printers consistently produced oversized 3D-printed models compared to the control and reported an error measurement of < 100 μm, demonstrating high trueness and clinically acceptable results [28]. We obtained similar results in the different groups when comparing the final volume of the printed models with the reference model. The studies that used orthodontic models had more relaxed thresholds for clinical acceptability (up to 500 μm) than those intended for prosthodontic applications (up to 200 μm) [28]. Accordingly, the choice of 3D printing technology should also be guided by its intended application.

As mentioned above, a standardised protocol for 3D printing of dental models is necessary to facilitate performance comparison involving all printing parameters, resins used, postprocessing protocol and time of assessment. In our study, we did not compare models printed with equal resolution, and we could not use equal layer thickness, *x*–*y* resolution or postprocessing due to the manufacturers and resin protocol followed, which could be a limitation. We selected 3D printers that are clinically applicable in the laboratories or industrial manufacturing centres as well as in the dental clinic due to their desktop format. The Multijet technology used in the industrial 3D printers implies bigger machines in volume and more expensive due to their building system, higher resolution and speed. We hope that the industry will research in this field in order to improve the technology in an affordable way that could be applied as a desktop printer and used in the dental field among others. This technological advance can also come with the current SLA and DLP desktop printers used in dentistry, improving their resolution and manufacturing properties.

To obtain the digital models used in the comparisons, both reference model and of the printed models, a state-of-the-art extraoral scanner has been used instead of a palpation CMM-type system. Although CMM-type palpation systems are the gold standard for calculating volumes of objects, these industrial systems were difficult to use with such a large sample size and with real patient models, since they use standard size probes that do not adapt correctly to all encountered surfaces. For this reason, the same extraoral scanner has been used in all the groups. The possible bias resulting from the measurement method should be the same in all the groups.

## Conclusions

Within the limitations of this study, there were statistically significant differences in terms of accuracy, trueness and precision, of the full-arch models of patients using five different 3D printers, both industrial and dental desktop printers. Multijet printing technology, which is normally used with industrial 3D printers, presented better results than the DLP and SLA technologies used in dental desktop 3D printers. A standardized protocol for 3D printing of dental models is necessary to facilitate performance comparison involving all printing parameters, the material used, the postprocessing protocol and the time of assessment.

## Data Availability

The research data is available through contact with the corresponding author and is registered at the Complutense University of Madrid.
